# Heterogeneity and causation of organ dysfunction in Alström syndrome

**DOI:** 10.1186/2046-2530-1-S1-P8

**Published:** 2012-11-16

**Authors:** R Paisey, C Carey, R Seymour, K Williams, C Rockett, K Vogler, S Bunce, A White, T Hiwot, R Cramb, M Waterson

**Affiliations:** 1 South Devon Healthcare NHS Foundation Trust, UK; 2 Queen Elizabeth Medical Centre, Birmingham, UK

## 

Alström syndrome (OMIM 203800) is an autosomal recessive condition caused by pathological mutations in the *ALMS1* gene of worldwide distribution prevalence. 200 subjects and longitudinal in depth studies of the UK cohort of 100 subjects have highlighted the extreme variation not only in the rate of visual and hearing loss but also in extent and progression of cardiac, renal, hepatic and endocrine dysfunction. The range in age at which visual acuity declines to <6/36 and/or when neuronal deafness is diagnosed is from 5 to 40 years. Life threatening cardiomyopathy occurs in 30% of neonates and can recur or arise de novo in 25% of all young adults from 12 to 25 years of age with sporadic cases later than this. Renal failure (CKD stage 5) occurs from 16 to 50 years in 25% of cases and hepatic cirrhosis in 10% between 10 and 40 years. Underlying this trend is some degree of cardiac and renal fibrosis and fatty liver in all, and severe insulin resistance. Progression from insulin resistance to type 2 diabetes and its severity and from fatty liver to fibrosis, then cirrhosis may be halted in early stages by lifestyle improvement especially in the second and third decades. Variation in deafness, renal and cardiac fibrosis differs within families but shows ethnic clustering suggestive that modifier genes contribute.

